# A comparative multi-level toxicity assessment of carbon-based Gd-free dots and Gd-doped nanohybrids from coffee waste: hematology, biochemistry, histopathology and neurobiology study

**DOI:** 10.1038/s41598-023-36496-4

**Published:** 2023-06-08

**Authors:** Halyna Kuznietsova, Natalia Dziubenko, Konstantin Paliienko, Natalia Pozdnyakova, Natalia Krisanova, Artem Pastukhov, Tetiana Lysenko, Marina Dudarenko, Valeriy Skryshevsky, Vladimir Lysenko, Tatiana Borisova

**Affiliations:** 1 Corporation Science Park, Taras Shevchenko University of Kyiv, 60 Volodymyrska Str., Kyiv, 01033 Ukraine; 2 Institute of High Technologies, Taras Shevchenko National University of Kyiv, Volodymyrska Street, 64, Kyiv, 01601 Ukraine; 3 Palladin Institute of Biochemistry National Academy of Sciences of Ukraine, 9 Leontovicha Street, Kyiv, 01054 Ukraine; 4 Light Matter Institute, UMR-5306, Claude Bernard University of Lyon/CNRS, Université de Lyon, 69622 Villeurbanne Cedex, France

**Keywords:** Transporters in the nervous system, Nanoparticles, Kidney diseases, Biomarkers, Inflammation

## Abstract

Here, a comparative toxicity assessment of precursor carbon dots from coffee waste (cofCDs) obtained using green chemistry principles and Gd-doped nanohybrids (cofNHs) was performed using hematological, biochemical, histopathological assays in vivo (CD1 mice, intraperitoneal administration, 14 days), and neurochemical approach in vitro (rat cortex nerve terminals, synaptosomes). Serum biochemistry data revealed similar changes in cofCDs and cofNHs-treated groups, i.e. no changes in liver enzymes' activities and creatinine, but decreased urea and total protein values. Hematology data demonstrated increased lymphocytes and concomitantly decreased granulocytes in both groups, which could evidence inflammatory processes in the organism and was confirmed by liver histopathology; decreased red blood cell-associated parameters and platelet count, and increased mean platelet volume, which might indicate concerns with platelet maturation and was confirmed by spleen histopathology. So, relative safety of both cofCDs and cofNHs for kidney, liver and spleen was shown, whereas there were concerns about platelet maturation and erythropoiesis. In acute neurotoxicity study, cofCDs and cofNHs (0.01 mg/ml) did not affect the extracellular level of L-[^14^C]glutamate and [^3^H]GABA in nerve terminal preparations. Therefore, cofNHs demonstrated minimal changes in serum biochemistry and hematology assays, had no acute neurotoxicity signs, and can be considered as perspective biocompatible non-toxic theragnostic agent.

Combination of imaging modality within the therapeutic entity is a promising approach to overcome the limitations of conventional treatments, which in turn ultimately improves the therapeutic efficacy. In this context, hybrid nanomaterials, nanohybrids (NHs), have attracted particular interests in biomedicine, owing to their distinctive physical and chemical properties^1^. A magnetic resonance imaging, MRI, is one of the most prevailing bioimaging diagnostics technique. Among lanthanides, the best contrast agent for MRI diagnostics^2^ is gadolinium (Gd) due to its high magnetic moment and longest electron spin relaxation time^3^, thereby providing the finest contrast MRI image. Incorporation of Gd to the nanostructures on the one hand can mitigate its toxicity in the organism, and on the other hand allows to use a phenomenon of nanoparticle accumulation in tumor. To overcome toxicity, Gd is usually embedded inside the nanovesicles (e.g. liposomes and micelles), metallic nanoparticles and carbon nanomaterials, etc.^4–9^.

Among carbon nanomaterials, the carbon dots (CDs) comprise a large diversity class of nanomaterials that is of special interest due to their low cost, simple production methods, low environmental impact and multidisciplinary application potential^10, 11^. A major advantage of CD production is the prospect to use a variety of precursors and methods^12^, and moreover CDs can be obtained in accordance with the green chemistry principles. The methodological approaches of their synthesis include microwave-assisted pyrolysis, electrochemical hydrothermal, solvothermal methods, etc.^13^. Also, according to the green chemistry principles, biowaste is widely used to produce CDs, in particular wheat straw^14^, rice residue^15^, fenugreek seeds^16^, fruit and vegetables peels^17–20^, sugar cane molasses^21^, coffee waste^22–26^, etc. CDs possess a defected composition of coexisting aromatic and aliphatic sites, the elementary constituents of which are graphene, graphene oxide and diamond, proportions and variations of which as well as variety of the groups at their surface depend on the original materials and the conditions of their synthesis.

CDs showed different biotoxicity in dependence on their precursors and production methods. In our previous study, neuroactive properties of CDs obtained from β-alanine by microwave heating were demonstrated. These nanoparticles (at high concentrations) affected key characteristics of inhibitory and excitatory, i.e. glutamate- and g-aminobutyric acid (GABA)-ergic, neurotransmission in isolated rat brain nerve terminals^27^. It should be underlined that glutamate and GABA are crucial excitatory and inhibitory neurotransmitters, respectively, in the central nervous system, impaired transport and homeostasis of which contribute to neuronal dysfunction and pathogenesis of major neurological disorders. In other study, it was revealed that sulfur-containing CDs synthesized from thiourea exhibit one third lower effects on glutamate and GABA transport in the nerve terminals in comparison with sulfur-free ones^28^.

Literature data showed that CDs synthesized from commercial synthetic chemicals can be dopped with Gd using energetically unfavorable hydrothermal treatment^29–32^ and adopted microwave-assisted method^33^. There is a lack of data on Gd-dopped carbon-based nanoparticle toxicity. In particular, the potential toxicity of Gd-doped CDs (Gd-CDs) synthesized via one-step solvent free technique with Gd-DTPA and L-arginine was assessed by serum biochemistry analysis. Gd-CDs demonstrated a low toxicity to the animals in a long-term application^34^. In other study, Gd-CDs were prepared using hydrothermal method with 3,4-dihydroxyhydrocinnamic acid, 2,2′-(ethylenedioxy)bis(ethylamine) and gadolinium chloride. Hemolysis assay of Gd-CDs showed no significant hemolysis phenomenon, thereby indicating a little damage to red blood cells and biocompatibility with the blood. In cytotoxicity study using human embryonic kidneys cells (293 T cells, CCK-8 assay), the cell viability was not changed by Gd-CDs that indicated their low cytotoxicity in vitro. Histopathology data of the treated mice tissues did not show apparent abnormalities or lesions in the heart, kidney, liver, and spleen, as compared to the control groups. It was concluded that Gd-CDs exhibited good biocompatibility and were suitable for further bioapplication^31^. Also, Gd meglumine was used along with citric acid and diethylenetriamine to synthesize Gd-CDs by a one-step hydrothermal method and the nanoparticles demonstrated inconspicuous cytotoxicity^32^. Gd-CDs conjugated with AS1411 aptamers were prepared via facile solvothermal approach. There was no evident damage in the 4T1 cells compared to the control group in in vitro assay, no obvious hemolysis was observed after nanoparticle application in vivo, and the synthesized nanoparticles exhibited good biocompatibility^35^.

As Gd per se possesses biotoxic features^36–38^, the question rose whether or not doping with Gd contributed to the nanoparticle toxicity. There are no literature data available on comparative toxicity of Gd-CDs and their precursor non-functionalized Gd-free ones.

Taking into account above mentioned facts, the aims of this study were: (*) a multi-level toxicity assessment of novel perspective ultrasmall carbon-based Gd-doped nanohybrids from coffee waste (cofNHs) obtained on the green chemistry basis, and (**) a comparison of cofNHs toxicity with their precursor, non-functionalized Gd-free CDs from coffee waste (cofCDs); using hematological, biochemical and histopathological methodological approaches after in vivo administration of the nanoparticles, and acute neurotoxicity study in vitro. The latter characterised the comparative effects of cofCDs and cofNHs on the crucial characteristic of glutamate- and GABA-ergic neurotransmission using nerve terminals isolated from rat cortex (synaptosomes), which are one of the best model systems to explore presynaptic processes^39^.

## Results

### Assessment of general toxicity of cofCDs and cofNHs

#### Gross toxicity and body weight change

**Figure 1:**
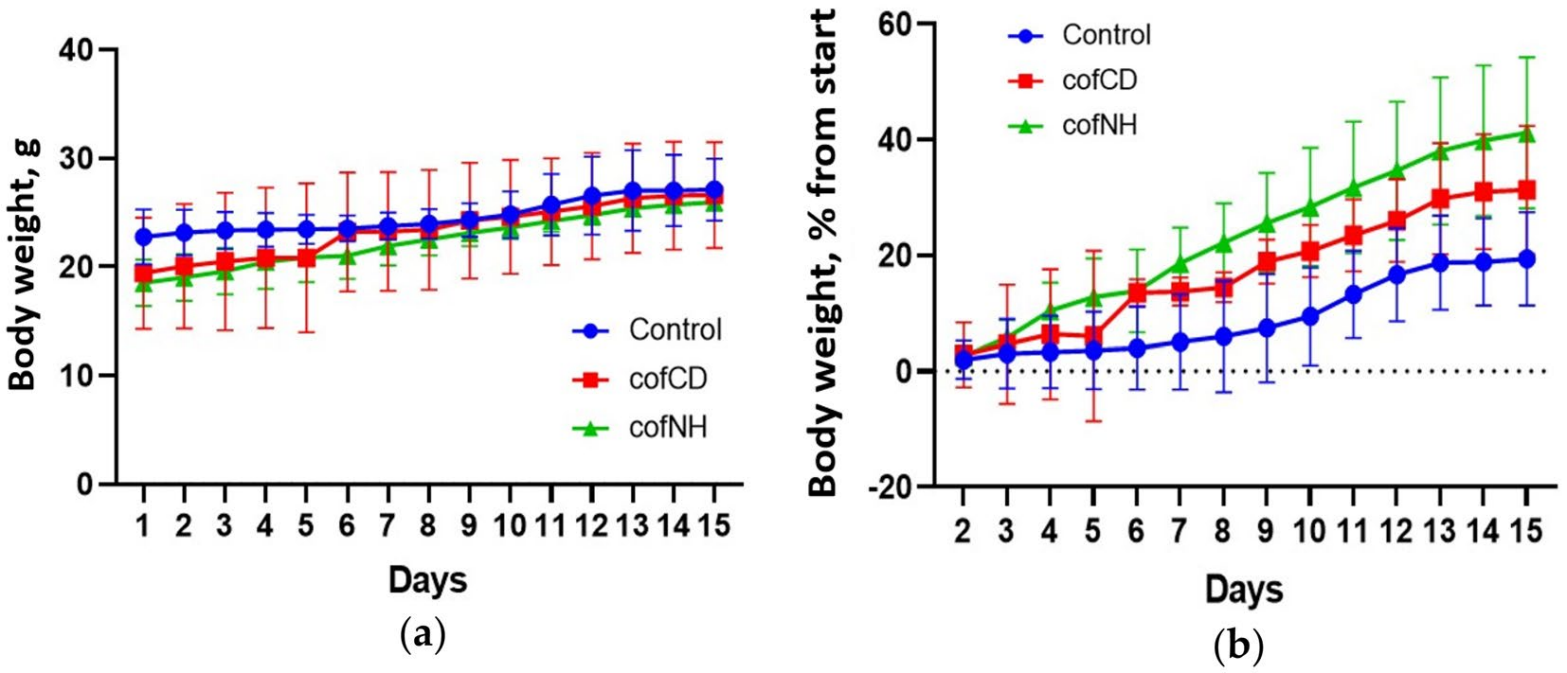
Body weight dynamics (absolute (**a**) and relative body weight changes (**b**)) of control mice and those treated with cofCDs and cofNHs for 14 days.

No toxicity was observed in any group of the experimental animals. Mortality also was not observed in the control and cofCD groups, however, 1 mouse died in cofNH group at the 6th day of the study. Furthermore, all the animals except that which died demonstrated consecutive body weight gain with no statistically significant difference between the groups, which could suggest mice wellbeing and no toxicity of the tested nanoparticles if applied in described doses for 14 days (Fig. 1).

#### Hematological assay

**Figure 2:**
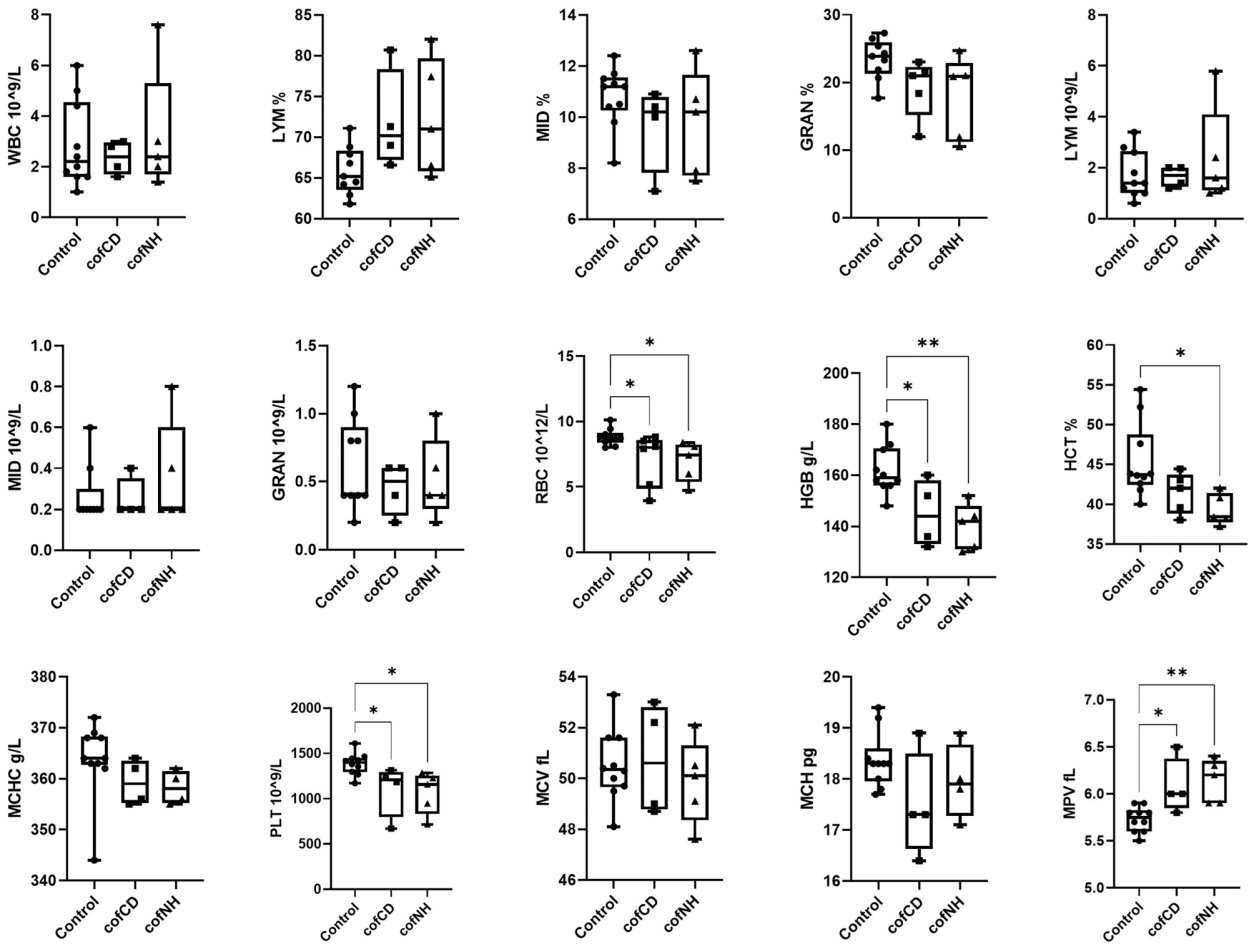
Hematological parameters of control mice and those treated with cofCDs and cofNHs for 14 days at the terminal day of the study. *p < 0.05, **p < 0.01.

According to the data shown in Fig. 2, both cofCDs and cofNHs application caused a tendency to increase in lymphocyte (LYM) percentage (*p* = 0.129 and *p* = 0.071, respectively) with concomitant decrease in neutrophilic granulocyte (GRAN) percentages (*p* = 0.179 and *p* = 0.063, respectively). These changes are usually an evidence of some specific inflammatory process in the organism, like viral or chronic bacterial infection, autoimmune disorders^40^. However, if there is no change in the absolute WBC count, we could assume rather a shift of immune response, but not a true inflammation. Increasing LYM% may take place in case of autoimmune disorders as a consequence of lymphocyte stimulation. Indeed, and CDs could cause such impact^41^. Therefore, we suggest that the observed changes might evidence some shift in immune response against the tested nanoparticles. However, we would like to notice that the observed values were still within the normal range typical for CD-1 mice^42^.

Erythrocyte (RBC) count and related parameters (hemoglobin (HGB), hematocrit (HCT), and mean corpuscular hemoglobin concentration (MCHC) values) demonstrated decreased values for both cofCDs and cofNPs in a similar manner. Decreased RBC, HGB, HCT and MCHC could evidence inhibition of erythropoiesis, and Gd probably does not contribute to this process. Then, increased mean platelet volume (MPV) with concomitant decrease of platelet (PLT) count in both groups could evidence the alteration of platelets maturation process, and, again, Gd is not supposed to be the main contributor to that. Taking together, there are some evidences of inflammation and violation of erythropoiesis and platelets formation, and it looks like that carbon core is the main contributor to these processes, and Gd adds no additional toxicity to that.

#### Biochemical assay

**Figure 3:**
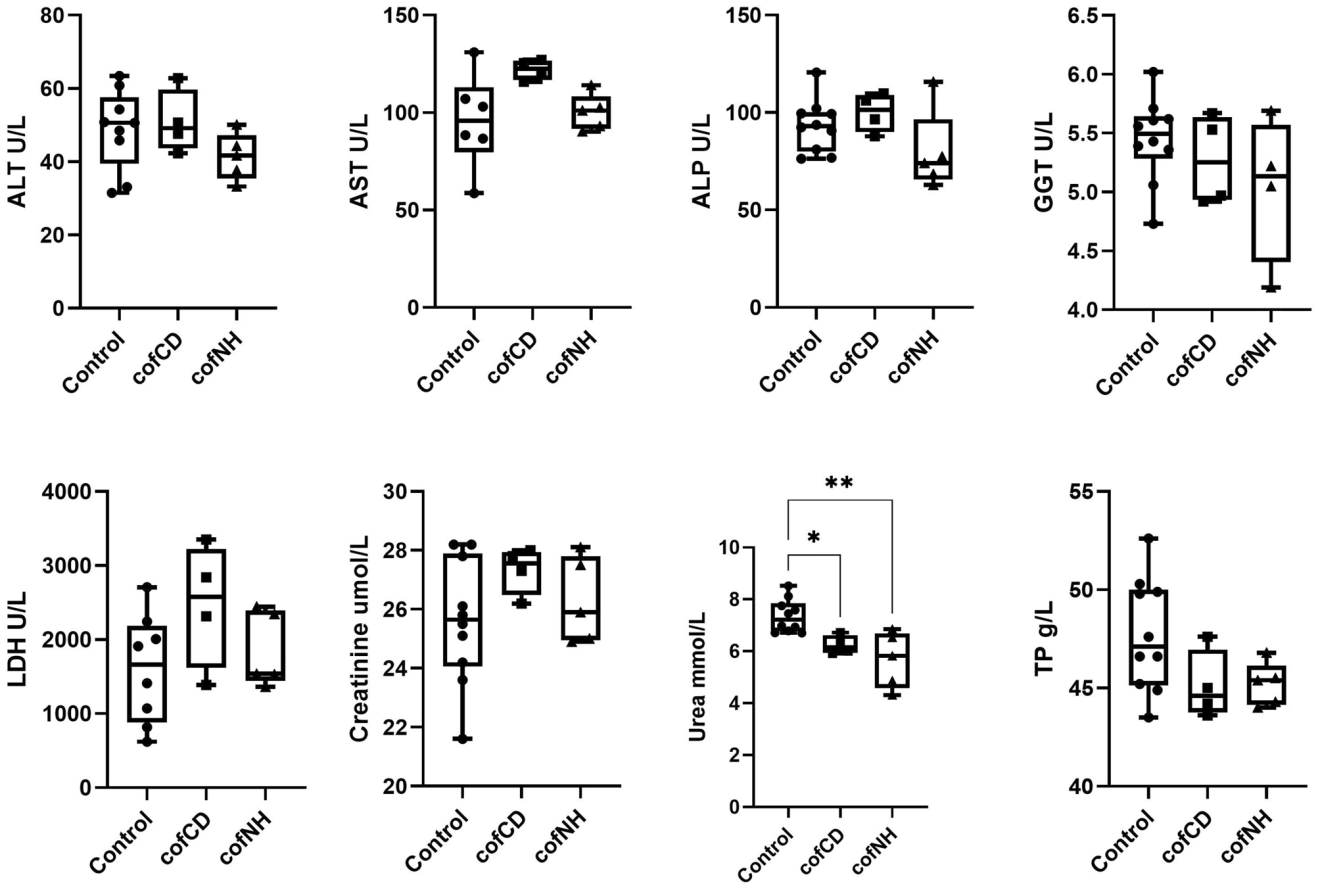
Serum biochemical parameters of control mice and those treated with cofCDs and cofNHs for 14 days at the terminal day of the study. **p < 0.01 compared to control.

According to the data presented in Fig. 3, there are no significant changes in liver enzymes’ activities, which could evidence no substantial impact of both cofCDs and cofNPs on liver function^43^. Urea’s decrease in cofCDs- and cofNH-treated groups together with a tend to decrease in total protein (*p* = 0.147 and *p* = 0.129, respectively). Such data might evidence protein deficiency in the organism due to poor protein intake or malabsorption, or some problems with protein synthesis and metabolism, which both take place in liver^44^. As the mice from all the groups continuously gained weight throughout the study (Fig. 1), therefore, malnutrition can be excluded. So, the reason of urea decrease in serum might be a consequence of impaired protein metabolism. It should be noted, however, that despite the significant changes compared to control, the values of serum urea were within the normal range, typical for CD-1 mice^42^.

Absence of serum creatinine concentration changes could evidence no impact on kidney function. Taking together, both cofCDs and cofNPs might lead to inhibition of protein synthesis, but these nanoparticles in applied doses caused no substantial violation of liver and kidney functions.

#### Histopathology assay

**Figure 4:**
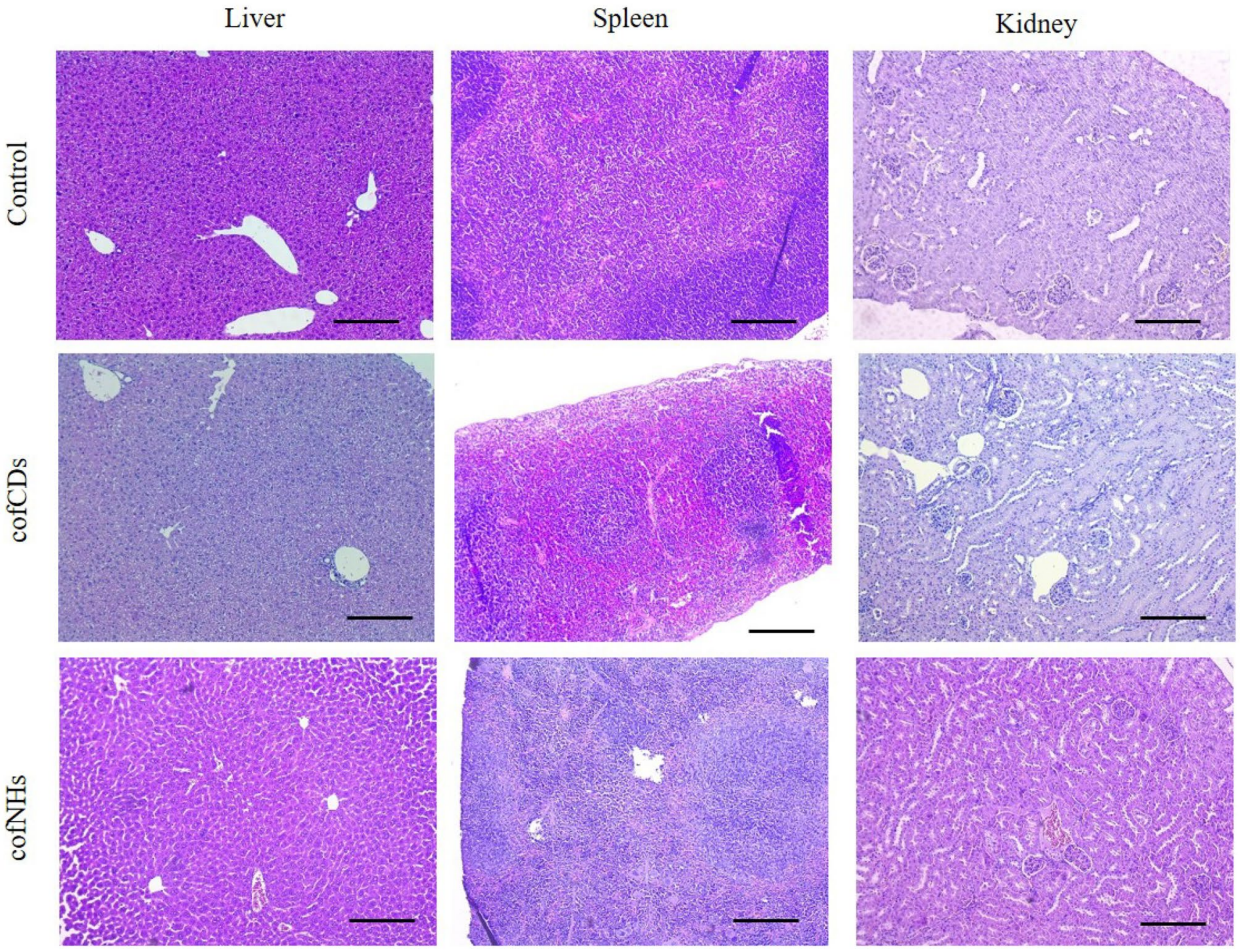
Microphotographs of kidney, liver and spleen of control mice and those treated with cofCDs and cofNHs. Magnification ×100, H&E. Scale 100 µm.

According to histopathological data presented in Tables 1, 2 and 3 and in Fig. 4, both cofCDs and cofNHs did not affect substantially liver, kidney and spleen states by that way which could be considered as the injury. However, there were still some structural changes. Thus, both cofCDs and cofNHs induced slight inflammatory signs in liver, manifested by slight Kupffer cell accumulation throughout the tissue, and occasional leukocytes accumulation loci, which is in line with our hematological findings. In cofNH-treated group blood vessel congestion sometimes also took place.

**Table 1:** Pathological changes of kidney of control mice and those treated with cofCDs and cofNHs.

Pathological changes	Control	cofCDs	cofNHs
Glomerulus shrinkage/capsule space dilation	−	−	−
Tubular epithelium flattening	−	−	−
Loss of brush border	−	+	+
Tubular epithelium loss	−	−	−
Tubular epithelium vacuolation	−	−	−
Tubular atrophy	−	−	−
Tubular dilation	−	−	−
Interstitial nephritis	−	−	−
Glomerulonephritis	−	−	−
Eosinophilic cast deposition	−	−	−
Necrotic loci	−	−	−
Hemorrhages	−	−	−
Vessel dilation	−	−	+
Fibrosis	−	−	−
Tubular basophilia	−	−	−
Tubular hyperplasia	−	+	+

Trait intensity: “−“—not observed, “+”—single or slight, “++”—moderate, “+++”—severe.

**Table 2:** Pathological changes of liver of control mice and those treated with cofCDs and cofNHs.

Pathological changes	Control	cofCDs	cofNHs
Steatosis	−	−	−
Hepatocellular hypertrophy	−	−	−
Vessel congestion	−	−	++
Lympho-histiocytes accumulation loci	−	+	−
Kupffer cell accumulation diffuse	−	+	+
Blood sinusoids dilation	−	−	−
Eosinophilic alteration	−	−	−
Basophilic alteration	−	−	−
Ground-glass hepatocytes	−	−	−
Necrotic hepatocytes	−	−	−
Apoptotic hepatocytes	−	−	−
Fibrosis (connective tissue accumulation)	−	−	−

Trait intensity: “−“—not observed, “+”—single or slight, “++”—moderate, “+++”—severe.

**Table 3:** Pathological changes of spleen of control mice and those treated with cofCDs and cofNHs.

Pathological changes	Control	cofCDs	cofNHs
Lymphoid hypoplasia (reduced white pulp)	−	−	−
Fibrosis (connective tissue accumulation)	−	+	−
Necrosis	−	+	+
Hemorrhages	−	−	−
Red pulp hyperplasia	−	−	−
White pulp hyperplasia	−	−	−
Megakaryocytosis	−	+	++
Marginal zone hyperplasia	−	+	+

Trait intensity: “−“—not observed, “+”—single or slight, “++”—moderate, “+++”—severe.

In kidneys, slight tubular epithelium loss of brush border was observed in both cofCD- and cofNH-treated groups, which, however, didn’t impact on kidney function as evidenced by serum creatinine and urea levels. Also, slight tubular hyperplasia took place in these two groups, which, despite occurring during chronic progressive nephropathy, is considered as the sign of tubular cells proliferation and tubule regeneration^45^. So, it could be concluded that kidneys were affected by tested compounds, but were regenerating successfully. Then, in cofNH-treated group blood vessel dilation sometimes took place, which is similar to the finding in liver, and could evidence some alteration of these organs’ blood supply.

Histopathological changes in spleen were similar in both cofCD- and cofNP-treated mice. Marginal zone hyperplasia (slight) and megakaryocytosis as increased number of megakaryocytes (from slight to moderate) were observed, as well as occasional necrotic loci. Marginal zone hyperplasia might evidence some activation of phagocytic system because of being populated by macrophages predominantly^45^. Our data about potential nanoparticle accumulation in spleen are in line with the literature^46, 47^. Megakaryocytosis is common in case of violation of platelet maturation and differentiation^48^, which confirms our suggestion based on hematological findings.

So, relative safety of both cofCDs and cofNHs for kidney, liver and spleen could be suggested. However, there were some concerns about platelet maturation and erythropoiesis, as well as slight inflammatory process in liver caused predominantly by carbon core. Nevertheless, these changes were minimal, so might not be an obstacle for multiple either cofCDs or cofNHs applications. Then, Gd strong capturing by cofCD core might be concluded because of no differences in biochemical, hematological and histopathological values in cofCD- and cofNH-treated groups.

### Assessment of acute neurotoxicity of cofCDs and cofNHs

#### Fluorimetric measurements of the membrane potential of nerve terminals after application of cofCDs and cofNHs

**Figure 5:**
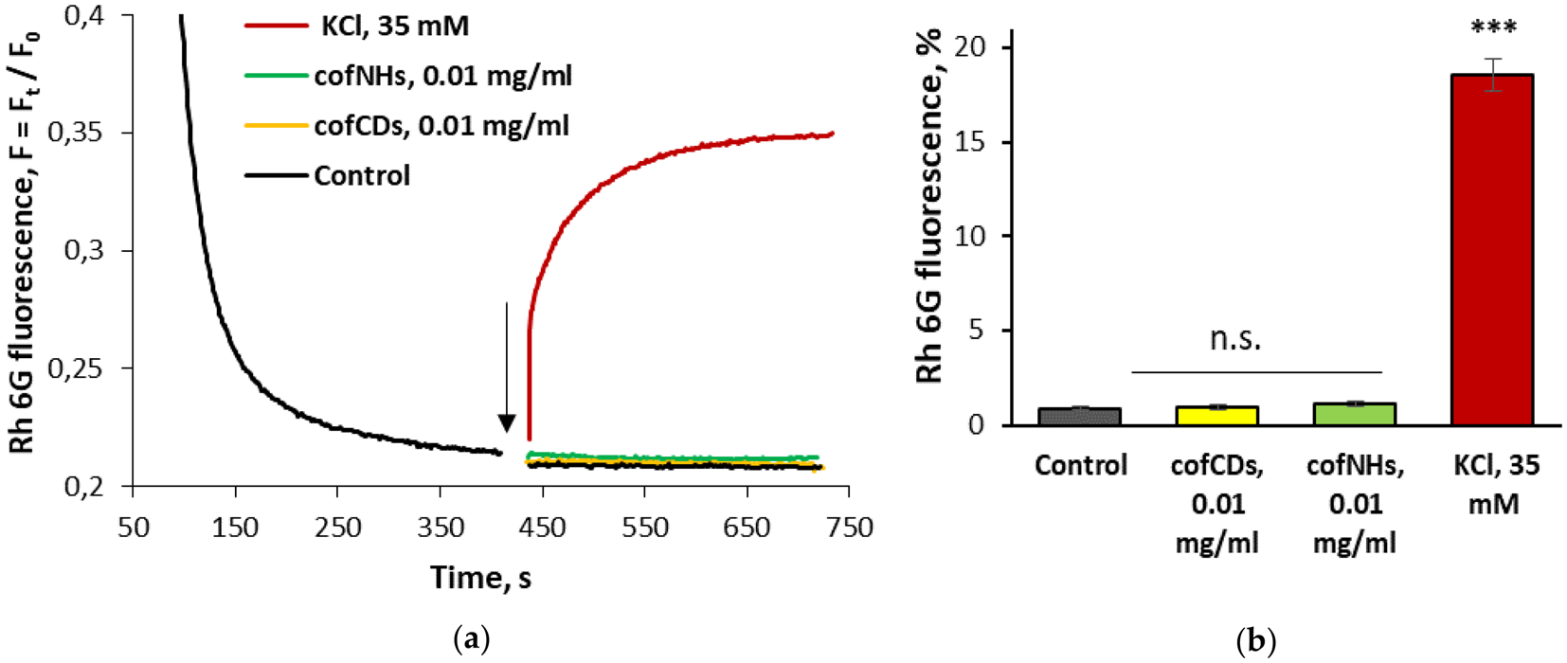
Fluorescence experiments: The membrane potential of nerve terminals in the presence of cofCDs and cofNHs. (**a**) The suspension of synaptosomes was equilibrated with potential-sensitive dye rhodamine 6G (0.5 mM); when the steady level of the dye fluorescence had been reached, the SSS (the control), cofCDs (0.01 mg/ml), cofNHs (0.01 mg/ml) and KCl (35 mM) (marked by the arrow) were added to synaptosomes. Traces are typical and represent 12 experiments performed with different synaptosome preparations. (**b**) An increase in the fluorescence signal of rhodamine 6G in response to application of cofCDs (0.01 mg/ml), cofNHs (0.01 mg/ml) and KCl (35 mM). Data are the mean ± SEM. ***, *p* < 0.001 as compared to the control; n.s., no significant differences; n = 12.

The membrane potential was monitored using the potential-sensitive fluorescent dye rhodamine 6G. F_st_, the membrane potential index at the steady state level, was achieved for 5 min, and it was set as 100% in statistical calculations. In fluorimetric experiments shown in Fig. 5a,b it was revealed that both cofCDs and cofNHs did not change the membrane potential of nerve terminals, and so did not depolarize their plasma membrane. To compare, the time-course of KCl (35 mM)-induced membrane depolarisation of nerve terminals was presented in Fig. 5.

#### The extracellular level of L-[^14^C]glutamate in nerve terminal preparations after application of cofCDs and cofNHs

Na^+^-dependent glutamate and GABA transporters are strategic players in the synaptic neurotransmission mediating neurotransmitter uptake to the cytoplasm of the presynaptic nerve terminals and establishing of the proper extracellular level of the neurotransmitters. The latter is a crucial synaptic parameter that represents a dynamic energy-dependent balance between the values of the transporter-mediated uptake and unstimulated leakage of the neurotransmitters^49, 50^.

As shown in Table 4, the extracellular level of L-[^14^C]glutamate in nerve terminal preparations was not changed by cofCDs and cofNHs at a concentration of 0.01 mg/ml.

**Table 4:** The extracellular level of L-[^14^C]glutamate in nerve terminal preparations after the application of cofCDs and cofNHs.

Nanoparticles	The extracellular level of L-[^14^C]glutamate in nerve terminal preparations (% of total accumulated label)	F;* p* value
Control without nanoparticles	21.96 ± 0.53	
cofCDs, 0.01 mg/ml	20.94 ± 0.44	F_(1,22)_ = 2.36; *p* = 0.13; n.s
cofNHs, 0.01 mg/ml	20.56 ± 0.57	F_(1,22)_ = 3.52; *p* = 0.07; n.s

Data were analysed using one-way ANOVA. Data are the mean ± SEM**.**, n.s., no significant differences compared to the control; n = 12.

#### The extracellular level of [^3^H]GABA in nerve terminal preparations after application of cofCDs and cofNHs

As shown in Table 5, the extracellular level of [^3^H]GABA in nerve terminal preparations was not changed significantly by cofCDs and cofNHs at a concentration of 0.01 mg/ml. These results are in accordance with above L-[^14^C]glutamate data. However, it should be noted that cofNHs had a strong tendency to increase the synaptosome extracellular level of [^3^H]GABA at this concentration.

**Table 5:** The extracellular level of [^3^H]GABA in nerve terminal preparations after application of cofCDs and cofNHs.

Nanoparticles	The extracellular level of [^3^H]GABA in nerve terminal preparations (% of total accumulated label)	F;* p* value
Control without nanoparticles	18.06 ± 0.43	
cofCDs, 0.01 mg/ml	18.85 ± 0.19	F_(1,22)_ = 3.03; *p* = 0.09; n.s
cofNHs, 0.01 mg/ml	19.17 ± 0.37	F_(1,22)_ = 4.18;. *p* = 0.053; n.s

Data were analysed using one-way ANOVA. Data are the mean ± SEM. n.s., no significant differences compared to the control; n = 12.

#### The extracellular level of L-[^14^C]glutamate and [^3^H]GABA in nerve terminal preparations after application of Gd^3+^

In the next series of the experiments, it was assessed the effects of Gd^3+^ on the extracellular level of L-[^14^C]glutamate and [^3^H]GABA in nerve terminal preparations. It was found that Gd^3+^ at concentrations of 1 and 10 µM Gd^3+^ related to its content in cofNHs did not change significantly the extracellular level of L-[^14^C]glutamate and [^3^H]GABA in nerve terminal preparations (Table 6). However, it should be noted that Gd^3+^ at a concentrations of 10 µM had a strong tendency to increase the synaptosome extracellular level of both L-[^14^C]glutamate and [^3^H]GABA.

**Table 6:** The extracellular level of L-[^14^C]glutamate and [^3^H]GABA in nerve terminal preparations after application of Gd^3+^.

	The extracellular level of L-[^14^C]glutamate in nerve terminal preparations (% of total accumulated label)	F;* p* value	The extracellular level of [^3^H]GABA in nerve terminal preparations (% of total accumulated label)	F;* p* value
Control	21.49 ± 0.75		11.91 ± 0.41	
Gd^3+^, 1 µM	21.95 ± 0.50	F_(1,22)_ = 0.28; *p* = 0.59; n.s	12.02 ± 0.65	F_(1,22)_ = 0.02; *p* = 0.88; n.s
Gd^3+^, 10 µM	23.71 ± 0.91	F_(1,22)_ = 3.87; *p* = 0.06; n.s	13.27 ± 0.58	F_(1,22)_ = 3.98; *p* = 0.058; n.s

Data were analysed using one-way ANOVA. Data are the mean ± SEM**.** n.s., no significant differences compared to the control; n = 12.

## Discussion

Here, a comparative assessment of general toxicity and acute neurotoxicity of cofCDs and cofNHs was performed in vivo and in vitro experiments, respectively. Serum biochemistry results revealed no changes in main liver enzymes, which could evidence no substantial toxicity against this organ, however, some inflammatory sings were observed. Decreased urea in both groups together with a tendency to decrease of TP could evidence protein synthesis inhibition. Taking together, both compounds in applied doses caused no substantial violation of liver and kidney functions. Hematology data demonstrated increased LYM% and concomitant decreased GRAN% in both groups that could evidence some inflammatory process in the organism and confirmed our histopathological findings in liver. Decreased (significant or as a tend) RBC, HGB, HCT and MCHC in cofCD- and cofNH- groups could evidence inhibition of erythropoiesis, and Gd barely contributed to that; increased MPV with concomitant decrease of PLT could evidence the alteration of platelets maturation process, which is in line with histopathological findings in spleen. Taking together, there were some evidences of inflammation and violation of erythropoiesis and platelets formation. As almost no differences in biochemical, hematological and histopathological values in cofNH-treated group were observed compared to cofCD-treated one, we could conclude that cofCD core likely contributes a lot to these processes.

Our experimental data on general toxicity of cofNHs are in accordance with the literature data. In particular, the potential toxicity of Gd-CDs and Gd-DTPA (widely used contrasting agent) was compared in mice injected with Gd-CDs (Gd concentration of 5 mg/kg) and Gd-DTPA via tail vein. In blood serum biochemistry analysis, the blood samples were collected during 1–21-day period. It was revealed that Gd-CDs exhibited similar effect as compared to Gd-DTPA. Analysis of the kidney indicators, blood urea nitrogen and creatinine did not reveal difference between the control and Gd-CDs group, thereby demonstrating absence of damage to the renal function. In hepatic function analysis, AST and ALT values were slightly increased after 1-day injection. Hepatic indicators, albumin and total protein, were maintained at the normal level. Gd-CDs and Gd-DTPA may affect the hepatic function shortly after the injection without significant damage of the hepatic tissues, because the liver could quickly recover its function after 7-day injection. Taking together, these results demonstrated a low toxicity of Gd-CDs and Gd-DTPA to the animals^34^. In other study, hemolysis was not revealed after administration of Gd-CDs (prepared using hydrothermal method with 3,4-dihydroxyhydrocinnamic acid, 2,2′-(ethylenedioxy)bis(ethylamine) and Gd chloride), thereby showing little damage to red blood cells and biocompatibility with blood. Histological analysis of the organs after 24 h post injection of Gd-CDs in toxicity study in vivo revealed no obvious damages in the Gd-CDs treated groups, such as inflammatory response, pulmonary fibrosis, necrosis or damages in major organs. Long term toxicity in vivo on healthy Kunming mice model for 16 days revealed that Gd-CDs did not induce obvious hepatic or kidney disorder in mice basing on the evaluation of TP, ALT, AST, ALP, blood urea nitrogen, total cholesterol, and triglyceride. Histopathology of treated with Gd-CDs mice revealed no apparent histopathological abnormalities or lesions observed in the heart, kidney, liver, and spleen, compared to the control. Any signs of necrosis were not revealed in the histological samples. In the lung tissues, peribronchial and perivascular cellular infiltrates were demonstrated that indicated moderate lung inflammatory responses. It was concluded that Gd-CDs exhibited good biocompatibility in vivo and can be suitable for further bioapplication^31^. No hemolysis phenomenon was observed studying the hemocompatibility of Gd-CDs conjugated with AS1411 aptamers (AS1411-Gd-CDs). It has been concluded that AS1411-Gd-CDs possessed the wonderful biocompatibility as for the application in biological field^35^.

In acute neurotoxicity study in vitro, cofCDs did not affect the ambient levels of L-[^14^C]glutamate and [^3^H]GABA in nerve terminal preparations at a concentration of 0.01 mg/ml. CofNHs did not affect the extracellular level of L-[^14^C]glutamate, but have a tendency to increase that of [^3^H]GABA at this concentration. Assessment of contribution of Gd revealed that Gd^3+^ ions at related concentrations demonstrated no significant changes, however had a tendency to increase the extracellular levels of both neurotransmitters in nerve terminal preparations. Despite cofNHs were not able to mitigate Gd neurotoxicity at this concentration (both are non-toxic), the presence of incorporated Gd in cofNH structure allow Gd to be accumulated in tumor in biomedical application based on tumor accumulating nanoparticle phenomenon. CofCD neurotoxicity results coincide with our previous experiments on CDs at related concentrations obtained from β-alanine and sulfur-containing CDs from thiourea and citric acid^27, 28^. In particular, CDs from β-alanine at concentrations of 0.01 mg/ml did not influence the extracellular level of L-[^14^C]glutamate and [^3^H]GABA in nerve terminal preparations^27^. Sulfur-containing CDs from thiourea and citric acid also had no effects on the synaptosome extracellular level of both neurotransmitters^28^.

Acute neurotoxicity data in vitro on CofNHs are in accordance with the literature data. In the cytotoxicity study in vitro using human embryonic kidneys cells (293 T cells) and CCK-8 assay, Gd-CDs (prepared using hydrothermal method with 3,4-dihydroxyhydrocinnamic acid, 2,2′-(ethylenedioxy)bis(ethylamine) and Gd chloride) even at a high concentration of 1 mg/mL did not change the cell viability after 24 h incubation that indicated their low cytotoxicity in vitro^31^. In other study, it was shown that Gd-CDs obtained by a one-step hydrothermal method had the inconspicuous cytotoxicity^32^. In particular, using NIH3T3 and 4T1 cells and CCK-8 assay, it was shown that the cells preserved high viability after 24 h coincubation, thereby indicating that Gd-CDs conjugated with AS1411 aptamers induced negligible toxicity^35^.

In perspectives, we plan to optimize the methodological approaches and perform acute neurotoxicity study at significantly higher (by 50 times) cofCD and cofNH concentrations to confirm or not whether tendency to increase in the extracellular [^3^H]GABA level in nerve terminal preparations by cofNHs (Table 4) resulted in significant increase in this parameter. This is so because a relatively low concentration of cofCDs and cofNHs was applied in vitro experiments (as compared to our previous CD-related study^27, 28^) due to untransparency and brown color of these nanoparticles. Nanoparticle-related concentrations of Gd^3+^ ions (Table 5) applied to the nerve terminals were also not high. Nevertheless, these concentrations of nanoparticles interrelated to cofCD and cofNH concentrations used in vivo animal study, and are applicable for potential MRI imaging in animals. Possible capability of cofNHs to increase the extracellular [^3^H]GABA level in nerve terminal preparations can be used as additional neurochemical theranostic feature. Theoretically, a compound that does not affect ambient glutamate level but increased the ambient GABA level in nerve terminals may possess antiepileptic, sedative and hypnotic effects.

## Materials and methods

### Materials

EGTA, EDTA, HEPES, Ficoll 400, Sigma-Fluor^®^ High Performance LSC Cocktail, the analytical grade salts were purchased from Sigma (USA); L-[^14^C]glutamate and [^3^H]GABA (**γ**-[2,3-3H(N)]-aminobutyric acid) were from Perkin Elmer (Waltham, MA, USA). Rhodamine 6G were obtained from Molecular Probes (USA).

### Synthesis of cofCDs and cofNHs

Microwave-assisted “green” synthesis of cofCDs and cofNHs from coffee waste was carried out according to a technique similar to that described in the study^51^ with additional purification stages. In particular, 5 g of coffee grouts were soaked with 0.1 M GdCl_3_ solution, dried, soaked with 10% NH_4_OH solution and sintered for 10 min in microwave oven in 250 ml round-bottom flask on air, making it possible to simultaneously proceed for ammoxidation reactions and interaction of hydrolyzed fragments with each other thanks to Maillard reactions, aldol condensation, alkylation of phenols, dehydration of the carbohydrates, etc. Gadolinium atoms may be retained by carboxylic groups as well as by hydroxyl groups of hydroxycinnamic acid derivatives abundant in coffee^52, 53^. Then, the nanoparticles were resuspended in distilled water, filtered through Vivaspin 20^®^ concentrators with polyethersulfone (PES) membranes of different pore sizes to get fraction lower than 30 kDa molecular weight, dialyzed through membrane of 3,500 MWCO (ZelluTrans ROTH^®^ Regenerated Cellulose Tubular Membrane), and preconcentrated using Vivaspin 20^®^ of 3,000 MWCO to get nanoparticles in range 3–30 kDa. Their physical and chemical properties were partially characterized^54^, as wells as TEM images (see Fig. S1) and FTIR spectra (Fig. S2) are provided in Supplementary Information.

### Ethics

#### In vivo study using animals

Female CD1 mice, 10–11 weeks old, with initial body weight of 19.6 ± 3.0 g were used in the study. Animals were kept in the animal facility of the Taras Shevchenko National University of Kyiv under natural lightning at 20–23 °C, and free access to standardized rodent diet and tap water. All experiments were conducted in compliance with bioethics principles, legislative norms and provisions of the European Convention for the Protection of Vertebrate Animals used for Experimental and Other Scientific Purposes^55^, General Ethical Principles for Experiments on Animals, adopted by the First National Bioethics Congress (Kyiv, 2001), and approved by the Institutional Animal Care and Use Committee (Protocol #1, June 24, 2021).

#### In vitro study using animals

Animals (Wistar rats, males, 12 weeks old, body weight of appx 120 g) were kept in the animal facilities of the Palladin Institute of Biochemistry, National Academy of Sciences of Ukraine, provided ad libitum with water and standardized rodent diet, and housed in a temperature-controlled room at 22–23 °C. Animal experiments were performed in accordance with the Guidelines of the European Community (2010/63/EU) and local laws/policies, and were approved by the Animal Care and Use Committee of the Palladin Institute of Biochemistry (Protocol # 1 from September 21, 2020). All animal studies were reported in accordance to the ARRIVE guidelines for reporting experiments involving animals^56, 57^. The total number of rats used in the study was 12, specifically, the measurements of the extracellular levels of L-[^14^C]glutamate and [^3^H]GABA in nerve terminals − 12 animals; and fluorimetry experiments shared these 12 animals.

### Toxicity study in vivo

#### Design of the study

Mice were randomly assigned onto 3 treatment groups (n = 5 in each) and received cofCDs and cofNHs dissolved in phosphate buffered saline (PBS) in concentration 25.0 mg/ml, or pure PBS (control group) intraperitoneally at the volume of 5 ml/kg (which corresponds to cofCDs and cofNHs doses of 125 mg/kg each) daily during 14 consecutive days, according to the recommendations for repeated dose toxicity studies for preclinical drug development^58, 59^. At the 15th day of the study, mice were anesthetized by 2,2,2-tribromoethanol (250 mg/kg) and sacrificed by cervical dislocation.

#### Examinations and observations

Mice general condition and body weight were monitored daily. The external state of the skin and fur, eyes, mucous membranes, the respiratory system, posture, and changes in spontaneous activity were evaluated. The detailed scoring system is presented in Table 7. Observations were performed immediately after the first administration, and once a day during the observation period.

**Table 7:** Gross toxicity scale.

Sign	Expression, score				
	0	1	2	3	4
General appearance	Normal		Unnatural posture/hunched posture	Emaciation	
Hypo/hyperkinesia	Absent	Decrease/increase activity	Drowsiness/aggression	unresponsive to extraneous activity and provocation	
Movement activity	Normal	Dysbasia/circling	Tremor	Convulsions, limb paralysis	
Respiration alterations	Absent	Deep/heavy/rapid/shallow			Respiratory arrest
Skin/coat injuries	Absent	Redness	Wounds	Abscess	Necrosis
Piloerection	Absent	Present			
Eyes conditions alterations	Absent	Pale/clouded/tearing	Sunken/inflame/half-closed	Closed eyes, do not open on touch	
Exudation	Absent		Ptyalism/nasal exudation		
Defecation changes	Absent	Abdomen abnormally enlarged/loose stool	Constipation	Diarrhea	Defecation with blood
Oedema/alterations including the site of administration	Absent	Oedema/other changes at the site of administration	Not at the site of administration		
Body temperature	Normal		Increased/decreased		
Vocalization	Absent	Occasional	Consistent		

#### Hematological assays

The blood for hematological analysis was collected immediately after the sacrifice by cardiac puncture, 25 µl of fresh blood was transferred into tubes with equal volume of 0.4% K_2_EDTA solution in saline. Assessment of the hematological parameters was performed using the hematology analyzer MCL-3124 (Guangzhou Mecan Trading Co., Ltd, China) and consumable reagents Cormay (Poland) within two hours after blood drawing. White blood cells count (WBC), lymphocyte (LYM), medium-sized cells (monocytes, eosinophils, and basophils, MID), neutrophils (GRAN) absolute and relative values, erythrocytes count (RBC), hemoglobin (HGB), hematocrit (HCT), platelet count (PLT) and average volume (MPV) were measured.

#### Biochemical assays

The blood for biochemical analysis was collected immediately after the sacrifice by cardiac puncture, left for 60 min to form a fibrin clot, and then centrifuged at 5400 g for 20 min at 4 °C. Blood serum was collected and used immediately for determination of the value of alanine aminotransferase (ALT), aspartate aminotransferase (AST), γ-glutamyl transpeptidase (GGT), lactate dehydrogenase (LDH), alkaline phosphatase (ALP), urea, creatinine, and total protein (TP). Analyses were performed on fully auto chemistry analyzer MF-240 (MedFuture LLC, USA) using standard reagent kits (Cormay, Poland) according to the protocols provided by manufacturer.

#### Histological assays

Liver, kidney, and spleen samples were harvested immediately after the sacrifice and fixed in 10% neutral buffered formalin for 7 days. After formalin fixation, the samples were dehydrated in ethanol solutions and embedded in paraffin, cut to obtain the slides of 5 µm thickness, which were deparaffinated and stained with hematoxylin and eosin (H&E) according to standard methods^60^, and examined under the light microscope by pathologist who was unaware of the treatment groups. Pathological features were assessed in a semi-quantitative manner, the detailed scoring systems are presented in Table 8.

**Table 8:** Organs’ toxicity scale*.

Feature	Indicators
*Kidneys*	
Glomerulus state	Glomerulus shrinkage/capsule space dilation
Tubular state	Epithelial cell flattening, epithelial cell vacuolation, epithelial cell desquamation/loss, loss of brush border, tubular atrophy, tubular dilation
Inflammation/necrosis signs	Eosinophilic cast deposition, tubular epithelial necrosis**, interstitial nephritis, glomerulonephritis, hemorrhages**, vessel dilation
Abnormal regeneration	Connective tissue accumulation, tubular basophilia, tubular hyperplasia
*Liver*	
Hepatocytes alteration	Lipid dystrophy, ground-glass hepatocytes, eosinophilic alteration, basophilic alteration
Regeneration	Hepatocellular hypertrophy, polyploid cells
Inflammation/necrosis signs	Vessel congestion/dilation, blood sinusoids dilation, lympho-histiocytes accumulation loci, Kupffer cell diffuse accumulation, necrotic loci**, apoptotic loci**
Connective tissue accumulation	
*Spleen*	
Hypo/hyperplasia	Lymphoid hypoplasia as reduced white pulp, lymphoid atrophy, red pulp hyperplasia, white pulp hyperplasia, megakaryocytosis, marginal zone hyperplasia
Inflammation/necrosis signs	Necrosis**, hemorrhages**
Accumulations	Lipid accumulation, pigmentation, connective tissue accumulation

*Trait intensity score: “−”—not observed or less than 10%; “+”—less than 50%; “++”—less than 80%; “+++”—more than 80% of field of view/number if counted.

**“−”—not observed; “+”—small occasional, “++”—small frequent, “+++”—large occasional, “++++”—large frequent.

Score results are presented below in the Result section.

### Acute neurotoxicity study in vitro

#### Isolation of nerve terminals (synaptosomes) from the cortex of the rat brains

The cortex brain region isolated from decapitated rats was immediately removed, and then homogenized in the ice-cold saline solution containing 0.32 M sucrose, 5 mM HEPES–NaOH, pH 7.4, and 0.2 mM EDTA. One synaptosome preparation was obtained from one rat, and each measurement was done in triplicate. The synaptosome preparations were obtained using differential and Ficoll-400 density gradient centrifugations of rat brain homogenate according to^61–63^. The synaptosome preparations were used in the experiments during 2–4 h. The standard saline solution (SSS) contained (in mM): NaCl 126; KCl 5; MgCl_2_ 2.0; NaH_2_PO_4_ 1.0; HEPES 20, pH 7.4; D-glucose 10. Protein concentration was examined according to^64^.

#### Measurements of the extracellular level of L-[^14^C]glutamate in the nerve terminal preparations

The synaptosome preparations were diluted in the SSS to reach a concentration of 2 mg of protein/ml, and after pre-incubation at 37 °C for 10 min were loaded with L-[^14^C]glutamate (2.81 μM, 1 µCi/ml) in the SSS at 37 °C for 10 min. After loading procedure**,** the synaptosome suspensions were washed with 10 volumes of ice-cold SSS; the pellets were re-suspended in the SSS to reach a final concentration of 1 mg of protein/ml. Synaptosome suspensions (125 μl; 0.5 mg of protein/ml) were pre-incubated at 37 °C for 10 min, then the aliquots of cofCDs and cofNHs were added and incubated with synaptosomes for 10 min, and then sedimented using a microcentrifuge (20 s at 10,000 g). The extracellular level of L-[^14^C]glutamate was recorded in the aliquots of supernatants (100 μl) and pellets using liquid scintillation counting with Sigma-Fluor^®^ High Performance LSC Cocktail (1.5 ml) and liquid scintillation counter Hidex 600SL (Finland), and the values were expressed as the percentage of total accumulated synaptosome L-[^14^C]glutamate^65, 66^. L-[^14^C]glutamate data were collected in triplicate from several (n) independent experiments performed with different synaptosome preparations.

#### Measurements of the extracellular level of [^3^H]GABA in the nerve terminal preparations

The synaptosome preparations were diluted in the SSS up to 2 mg of protein/ml, and after their pre-incubation at 37 °C for 10 min were loaded with [^3^H]GABA (50 nM, 4.7 µCi/ml) in the SSS for 10 min. GABA transaminase inhibitor aminooxyacetic acid at a concentration of 100 µM was used during [^3^H]GABA loading and release experiments to minimize the formation of GABA metabolites. After loading, the synaptosome suspensions were washed with 10 volumes of ice-cold SSS. The pellets were re-suspended in the SSS to have protein concentration of 1 mg/ml. Synaptosome suspensions (120 µl) were pre-incubated at 37 °C for 10 min, then the aliquots of cofCDs and cofNHs were added and incubated for 10 min, and sedimented using a microcentrifuge (20 s at 10,000 g). The extracellular level of [^3^H]GABA in synaptosome preparations was recorded. [^3^H]GABA radioactivity was measured in the aliquots of supernatants (90 µl) by liquid scintillation counting with Sigma-Fluor^®^ High Performance LSC Cocktail (1.5 ml) and liquid scintillation counter Hidex 600SL (Finland), and the values were expressed as the percentage of total accumulated synaptosome [^3^H]GABA^67^. [^3^H]GABA data were collected in triplicate from several (n) independent experiments performed with different synaptosome preparations.

#### The synaptosome membrane potential (Em)

The membrane potential of synaptosomes in the presence of cofCDs and cofNHs was measured using the potentiometric fluorescent dye rhodamine 6G (0.5 µM) based on its potential-dependent binding to the membranes^68–70^. The synaptosome suspension (a final concentration of 0.2 mg of protein /ml) were preincubated at 37 °C for 10 min, and then added to a thermostated cuvette with continuous stirring. The synaptosome suspension was equilibrated with the probe, and the aliquots of cofCDs and cofNHs were added. To estimate changes in the plasma membrane potential the ratio (F) as an index of membrane potential was calculated according to Eq.: F = F_t_/F_0_, where F_0_ and F_t_ are fluorescence intensities of a fluorescent dye in the absence and presence of the synaptosomes, respectively. F_0_ was calculated by extrapolation of exponential decay function to t = 0. Fluorescence measurements with rhodamine 6G were carried using a Hitachi MPF-4 spectrofluorimeter at 528 nm (excitation) and 551 nm (emission) wavelengths (slit bands 5 nm each).

#### Statistical analysis

GraphPad Prism 9.0.0 software was used for statistical analysis and data visualisation. Homogeneity of variance was assessed using the Levene test. The experimental data were expressed as the mean ± S.E.M. of *n* independent experiments. The difference between two groups was compared by one-way analysis of variance (ANOVA) with the Tukey post hoc test. Mann–Whitney *U-*test for independent samples was used for analysis of histopathological signs scores. Differences were considered significant, when *p* < 0.05.

## Limitations of the study

Short-term toxicity study (14 days of administration) was performed, which does not allow to make a strict conclusion about no delayed toxicity of the tested chemicals. However, as the main purpose of Gd-dopped nanomaterials is bioimaging application, i.e. single administration, the terms used in this toxicity study allow at least to exclude acute toxicity of the nanoparticles, and therefore could be a basis for conducting preclinical animal research of these chemicals during long-term administration.

## Conclusions

Summarizing, a comparative multi-level toxicity assessment showed relative safety of both cofCDs and cofNHs for kidney, liver and spleen. There were some concerns about platelet maturation and erythropoiesis, as well as potential affection of liver, but Gd incorporation was unlikely related to that. In total, cofNHs have demonstrated minimal changes in serum biochemistry and hematology assays that are not an obstacle for biomedical application of cofNHs. Also, cofCDs and cofNHs did not influence the extracellular levels of L-[^14^C]glutamate and [^3^H]GABA in nerve terminal preparations, and so had no acute neurotoxicity signs. Taking together, it may be considered that cofNHs can be further analyzed in biomedical research as perspective theragnostic agent.

## Supplementary Information

Supplementary Information.

## Data Availability

The datasets used during the current study are available from the corresponding author upon reasonable request. Partially, data on nanoparticle synthesis are available in the Proceedings of the C’Nano 2023: The Nanoscience Meeting; Poitiers, 2023; p. 24.
