# Supplementary material for: A comparative multi-level toxicity assessment of carbon-based Gd-free dots and Gd-doped nanohybrids from coffee waste: hematology, biochemistry, histopathology and neurobiology study
Source: Sci Rep. 2023 Jun 8;13:9306. doi: 10.1038/s41598-023-36496-4 (PMC10250545; doi:10.1038/s41598-023-36496-4)
Supplement: Supplementary file 1 — Supplementary Information. [file 41598_2023_36496_MOESM1_ESM.docx]

**Supplementary Information**

A comparative multi-level toxicity assessment of carbon-based Gd-free dots and Gd-doped nanohybrids from coffee waste: hematology, biochemistry, histopathology and neurobiology study

^1^ Corporation Science Park, Taras Shevchenko National University of Kyiv, 60 Volodymyrska Str., Kyiv, 01033, Ukraine 1; [skrysh@univ.kiev.ua](mailto:skrysh@univ.kiev.ua)

^2^ Institute of High Technologies, Taras Shevchenko National University of Kyiv, Volodymyrska Street, 64, 01601 Kyiv, Ukraine; [skrysh@univ.kiev.ua](mailto:skrysh@univ.kiev.ua)

^3^ Palladin Institute of Biochemistry National Academy of Sciences of Ukraine, 9 Leontovicha Street, Kyiv, 01054, Ukraine; [tborisov@biochem.kiev.ua](mailto:tborisov@biochem.kiev.ua)

^4^ Light Matter Institute, UMR-5306, Claude Bernard University of Lyon/CNRS, Université de Lyon, 69622, Villeurbanne Cedex, France; [vladimir.lysenko@univ-lyon1.fr](mailto:vladimir.lysenko@univ-lyon1.fr)

***** Correspondence: kostyaram@gmail.com

**

**

**Figure S1**. Typical TEM image of cofNHs sample, X200k magnification. Nanoparticles water solution was deposited on the surface of ultrathin carbon film CF300-CU-UL 50/bx and dried by filter paper on air. Microscopy was done on TEM Jeol 1400 Flash, 120 kV. On the photo one can observe ultra-small amorphous structures 4-8 nm in diameter.


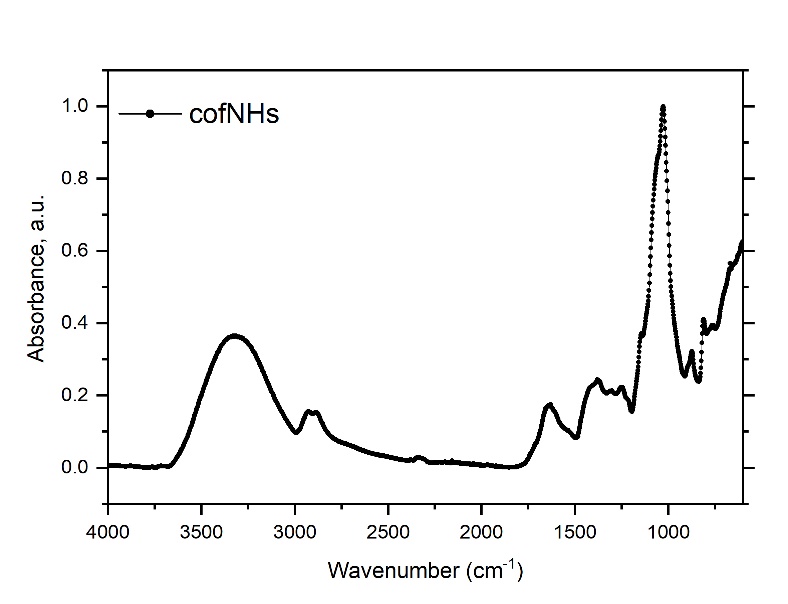


**Figure S2**. FTIR-ATR spectrum of the lyophilized sample cofNHs sample. It was performed on IRAffinity-1 Shimadzu^®^ IR-spectrometer in Happ-Genzel apodization mode. The wide band at 3400-3000 cm^-1^ corresponds to stretching vibrations ν(O–H) of hydroxyls, medium-intensity bands at 3000-2800 cm^-1^ to ν(C–H) of sp^3^-hybridized alkane bonds, and the most intense peak at 1024 cm^-1^ – to ν(C–O) of carbonyls, 1650 cm^-1^ could be referred to: asymmetrical stretches of ionic or Gd-coordinated carboxylate anions ν_s_(–CO^2–^), C=O stretches of amides or other conjugated species, C=C stretches in aromatic rings, etc. The band at 1544 cm^-1^ could be referred to C=N stretches in conjugated systems or to amide-II vibrations in polypeptide fragments. The band between 1420 and 1380 cm^-1^ belongs to deformation vibrations of C–H and O–H bonds in organic species and to deformations of NH^4+^-cation, also due to symmetrical stretches of the carboxylate ν_s_(–CO^2–^) and some vibrations of aromatic fragments.
